# Supplementary figures and images for: Challenging Safety and Efficacy of Retinal Gene Therapies by Retinogenesis
Source: Int J Mol Sci. 2021 May 28;22(11):5767. doi: 10.3390/ijms22115767 (PMC8198227; doi:10.3390/ijms22115767)

## Supplementary Figure S1

**A**

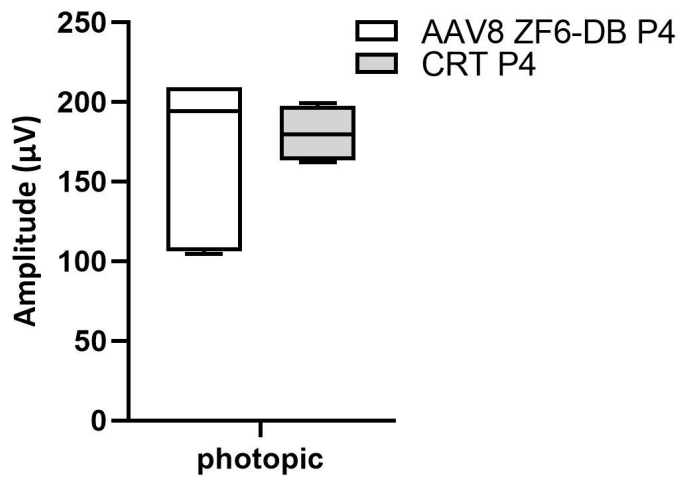

**B**

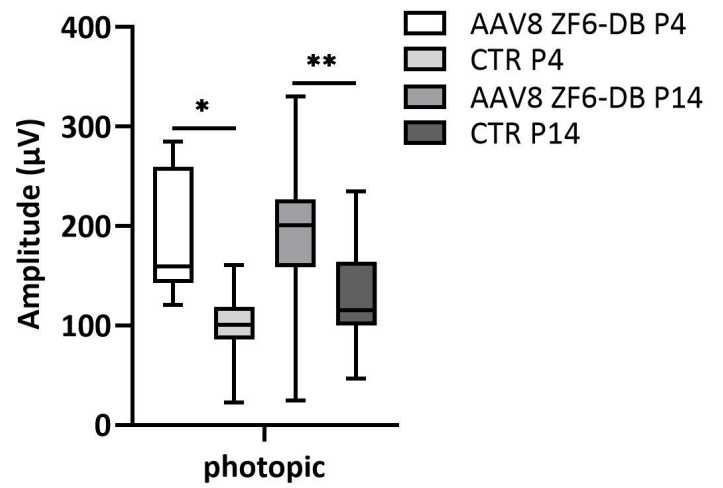

## Supplementary Figure S2

**A**

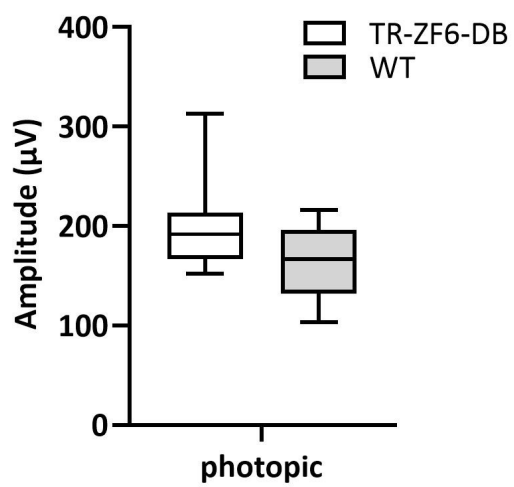

**B**

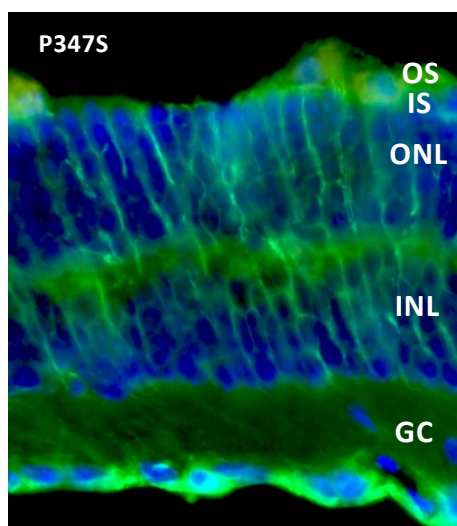

**C**

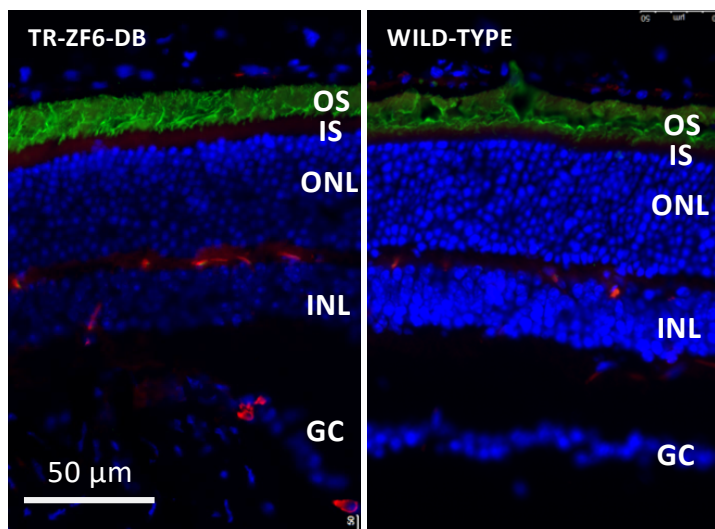

Supplementary Figure S3

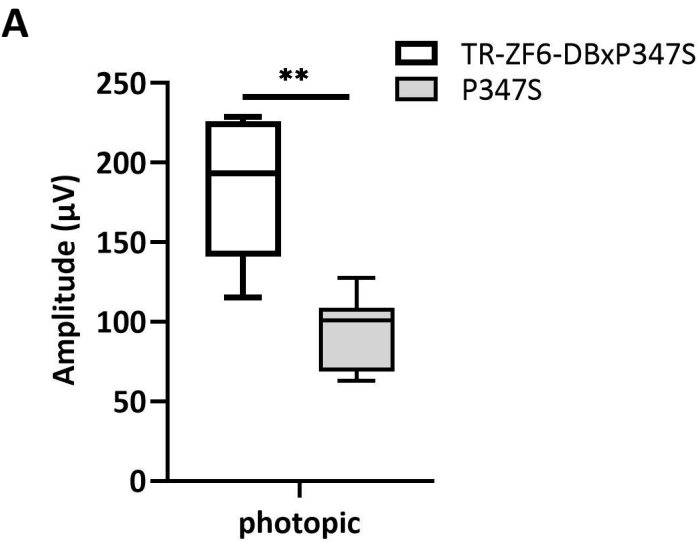

Supplement: Supplementary file 1 [file ijms-22-05767-s001.zip › ijms-1212284-supplementary.pdf]
